# Comparison of Red Blood Cell Features in Israeli Circassians: The Possible Effect of Residential Altitude

**DOI:** 10.5041/RMMJ.10574

**Published:** 2026-04-26

**Authors:** Leonid Livshits, Carina Levin, Galina Petukhova, Snait Ayalon, Sireen Sharif, Ariel Koren

**Affiliations:** 1 Faculty of Sciences, Tel Hai University of Kiryat Shmona in the Galilee, Kiryat Shmona, Israel; 2 MIGAL Galilee Research Institute, Kiryat Shmona, Israel; 3 Pediatric Hematology Unit, Emek Medical Center, Afula, Israel; 4 The Bruce and Ruth Rappaport Faculty of Medicine, Technion–Israel Institute of Technology, Haifa, Israel; 5 Research Authority, Emek Medical Center, Afula, Israel

**Keywords:** Anemia, hemoglobin, red blood cells, residential altitude

## Abstract

**Background:**

The Circassian community in Israel represents a unique, endogamous population residing in two villages in the northern region of Israel: Kfar Kama (209 meters above sea level [m.a.s.l.]) and Rihaniya (674 m.a.s.l.).

**Objectives:**

This study sought to investigate whether the small difference in altitude between these two locations (465 m) affects red blood cell (RBC) and hemoglobin (Hb) parameters in the residents.

**Methods:**

We examined the data from 2,517 blood samples collected from adult Israeli Circassians over 2.5 years (January 2020–June 2022). To ensure independent observations, analysis was limited to each participant’s most recent complete blood count (CBC). Subjects were stratified by sex, age, anemia status, and residential location.

**Results:**

Our analysis revealed that a difference of about 465 meters in residential altitude significantly elevated Hb, RBC count, and hematocrit (HCT) levels in non-anemic male and female cohorts. This elevation was accompanied by a small but significant decrease in mean corpuscular volume (MCV) and mean corpuscular hemoglobin (MCH). Furthermore, we found that the effect of altitude on most RBC characteristics differed in anemic individuals of both sexes, although their RBC count still increased.

**Conclusions:**

Even minor differences in residential altitude can significantly influence Hb and RBC parameters. This observation appears only in non-anemic individuals, probably reflecting physiological altitude Hb adaptation mechanisms. While further research is needed to fully elucidate the underlying mechanisms, our findings provide new data on the physiological impact of altitude on human health, particularly at low altitudes.

## INTRODUCTION

The Circassians (also known as Adyghe [Aдыгэxэp]) are one of the oldest indigenous peoples of the North Caucasus. Historically, they were composed of twelve major tribes, each with a distinct identity yet sharing a common language and culture. Following their resettlement in the Ottoman Empire during the 19th century, Circassians established small communities in Transjordan, the Golan Heights, and Syria. Despite more than 150 years having passed since their displacement, they have remarkably preserved their unique ethnic identity, culture, and traditions.^1^,^2^

In Israel, the Circassian population numbered 4,638 people in 2023. They live in relative cultural and geographical isolation in two distinct villages in the northern part of the country: Kfar Kama in the Lower Galilee (3,455 residents) and Rihaniya in the Upper Galilee (1,183 residents). The residents of Kfar Kama are descendants of the Shapsug tribe, while those in Rihaniya are from the Abzakh tribe. Endogamous marriages across the global Circassian population have played a significant role in maintaining a distinct genetic profile of the community. Hence, this community is a unique subject for population-based biological research, despite the scarcity of existing medical or genetic studies on Middle Eastern Circassians.^2^–^4^

Israel’s topography is unique, with extreme altitude changes over a limited area. The villages of Kfar Kama and Rihaniya are only about 44 km apart, but their altitudes differ significantly, at 209 and 674 meters above sea level (m.a.s.l.), respectively. This study investigated whether this relatively small difference in altitude affects red blood cell (RBC) and hemoglobin (Hb) parameters among residents.

Numerous studies have shown a direct correlation between an individual’s permanent residential altitude and their blood Hb concentration, with an average increase of 0.3–1.1 g/dL per 1000 meters of elevation in moderate and high altitudes.^5^–^8^ Moreover, this effect has also been observed at lower elevations.^9^–^12^ However, these studies typically compare data from residents living in geographically distant locations. Furthermore, it is well established that ethnic and racial characteristics can significantly influence Hb levels and other RBC features.^5^,^7^,^13^–^15^ The specific examination of this phenomenon within one, well-preserved, homogenic ethnic group may provide a precise view of the physiological impact of adaptation to low to moderate elevations.

## METHODS

### Study Design and Population

This retrospective cohort study used the electronic health record database of Clalit Health Services (CHS). The CHS is an Israeli payer-provider integrated health maintenance system (HMO), serving >4.5 million members, constituting 54% of the Israeli population. The CHS HMO database securely stores all patient data including demographic and clinical characteristics, hospital discharge and outpatient clinic diagnoses, and laboratory test results. Data were extracted from CHS using its research data-sharing platform powered by MDClone (https://.mdclone.com). The dataset for our study, the data dictionary, was used with permission; complete documentation is securely held by the CHS HMO and, due to ethical restrictions, cannot be shared.

Data spanning 2.5 years (January 2020 to June 2022) were included in the analysis. Blood counts performed when the subjects were examined at hospital emergency rooms or during hospitalization were excluded in order to obtain maximal “steady state” results. In total, 2,517 individual blood samples were analyzed. To ensure independent observations, analysis was limited to each participant’s most recent complete blood count (CBC). Data were then subdivided based on sex, age, and anemia status. Anemia was defined as Hb level <12 g/dL for women and Hb <13.5 g/dL for men).^16^

### Ethics Approval

This study was approved by the ethics committee of the Emek Medical Center (EMC-0087-22) in keeping with the principles of the Declaration of Helsinki. In accordance with Israeli Ministry of Health regulations, the institutional ethics committee did not require written informed consent, since data were collected anonymously from computerized medical files and there was no active patient participation.

### Laboratory Testing

All laboratory analyses were performed by CHS-licensed medical laboratories, in strict compliance with International Organization for Standardization (ISO) standards. Samples exhibiting partial or missing results in CBC tests were excluded from subsequent analysis.

### Statistical Analysis

Descriptive and inferential statistical analyses were performed to examine differences between groups from two villages (Kfar Kama and Rihaniya) using the available datasets. Continuous variables were summarized using means and standard deviations, while categorical variables were presented as frequencies and percentages. To assess the effect of residential altitude on hematologic indices, we employed multivariable linear regression models with high goodness-of-fit (coefficient of determination; *R^2^*≥0.992). These models included interaction terms to evaluate whether the association between village locations and hematological outcomes was modified by age, sex, or anemia status. Statistical analysis was carried out using R software version 4.1.3 (R Core Team, 2022; R Foundation for Statistical Computing, Vienna, Austria) and RStudio 2022.07.1 Build 554 (Posit Software, PBC, Boston, MA, USA). Statistical significance was set at *P*≤0.05.

## RESULTS

We analyzed the CBC data collected from the two Circassian villages in Israel: Kfar Kama (1,927 samples; 209 m.a.s.l.) and Rihaniya (698 samples; 674 m.a.s.l.). The total number of samples reflected a significant percentage of the adult population in both villages.

Across the whole cohort, our analysis revealed no significant elevation in Hb levels in female and male residents of Rihaniya compared to those in Kfar Kama. The RBC count was significantly higher, and the mean corpuscular volume (MCV) and mean corpuscular hemoglobin (MCH) were significantly lower in both sexes in Rihaniya compared to the Kfar Kama population (Table 1). However, hematocrit (HCT), which reflects both RBC count and MCV, was non-significantly elevated for both female and male cohorts in both villages. Mean corpuscular hemoglobin concentration (MCHC) and red cell distribution width showed small but significant related fluctuations in the male population only. The Hb trends observed in the general population persisted across most age groups (Table 2), although these comparisons did not reach statistical significance.

**Table 1:** Red Blood Cell Parameters of Male and Female Participants Categorized by Their Residence.

Sex	Place of Residence	*N*	Hb (g/dL)	RBC (10^6^/μL)	HCT (%)	MCV (fL)	MCH (pg)	MCHC (g/dL)	RDW (%)
Female	Kfar Kama	1076	12.49±1.15 (7.70–17.00)	4.41±0.42 (2.33–6.40)	38.07±3.37 (22.70–52.00)	86.53±6.12 (60.70–119.90)	28.41±2.23 (17.40–38.60)	32.83±1.04 (28.20–36.80)	13.87±1.41 (11.10–23.00)

	Rihaniya	373	12.79±1.20 (7.40–17.20)	4.59±0.42 (3.16–6.31)	38.88±3.39 (25.60–53.20)	84.97±7.04 (58.30–98.90)	27.98±2.74 (16.80–33.20)	32.90±1.24 (28.40–37.00)	13.77±1.47 (11.80–22.60)
	β		0.06 (0.06)	0.21 (0.03)	0.28 (0.19)	−3.14 (0.42)	−1.08 (0.15)	−0.12 (0.07)	0.16 (0.08)
	*P*		0.305	**<0.001**	0.144	**<0.001**	**<0.001**	0.124	0.055

Male	Kfar Kama	779	14.56±1.39 (5.70–18.10)	4.97±0.50 (2.16–7.20)	43.79±4.01 (17.80–53.70)	88.42±5.23 (62.40–110.30)	29.40±1.88 (20.00–35.30)	33.25±1.12 (29.20–37.00)	13.51±1.18 (11.50–24.40)

	Rihaniya	289	14.67±1.46 (7.60–18.70)	5.12±0.51 (3.64–7.52)	44.18±4.01 (27.20–55.90)	86.70±7.08 (57.50–109.70)	28.80±2.65 (18.80–35.50)	33.20±1.27 (27.90–36.70)	13.56±1.16 (11.20–18.90)
	β		−0.01 (0.08)	0.24 (0.03)	0.21 (0.25)	−3.79 (0.55)	−1.40 (0.20)	−0.23 (0.10)	0.28 (0.11)
	*P*		0.898	**<0.001**	0.406	**<0.001**	**<0.001**	**0.025**	**0.015**

Data presented as means±SD (range). Regression coefficients β (with standard error) were derived from a multivariable linear regression model adjusted for the interaction between sex and residence (village). Statistical significance was set at *P*≤0.05 and is marked in bold.

Hb, hemoglobin; HCT, hematocrit; MCHC, mean corpuscular hemoglobin concentration; MCV, mean corpuscular volume; *N*, number of participants; RBC, red blood cell count; RDW, red cell distribution width.

**Table 2:** Mutual Influence of Age and Residential Altitude on Hb Concentration of Male and Female Participants.

Age (years)	Place of Residence	Female		Male	

		Number	Hb (g/dL)	Number	Hb (g/dL)
18–30	Kfar Kama	265	12.43±1.02 (8.40–15.10)	137	15.01±0.88 (12.50–17.30)
	Rihaniya	84	12.65±1.06 (8.30–15.00)	59	15.23±1.09 (12.80–18.70)

31–40	Kfar Kama	203	12.43±1.08 (7.80–14.90)	132	14.82±1.01 (10.10–17.70)
	Rihaniya	81	12.56±1.34 (7.90–14.90)	41	15.03±0.89 (12.90–17.40)

41–50	Kfar Kama	183	12.60±1.10 (8.50–15.20)	121	14.84±1.15 (11.00–18.00)
	Rihaniya	61	13.1±0.95 (10.60–16.00)	50	15.03±1.21 (12.10–17.70)

51–60	Kfar Kama	129	12.87±1.09 (9.60–17.00)	142	14.83±1.38 (8.70–18.10)
	Rihaniya	51	12.89±1.07 (9.20–15.40)	52	14.43±1.39 (10.40–17.40)

61–70	Kfar Kama	150	12.77±1.08 (9.20–15.90)	149	14.17±1.42 (5.70–16.80)
	Rihaniya	58	13.10±1.29 (8.70–17.20)	43	14.66±1.39 (9.10–18.10)

71–80	Kfar Kama	106	12.24±1.34 (7.70–15.30)	70	13.85±1.68 (8.10–17.50)
	Rihaniya	29	12.58±1.44 (7.40–14.70)	32	14.00±1.59 (10.00–16.20)

>80	Kfar Kama	43	11.31±1.38 (8.70–13.40)	28	12.35±1.89 (8.90–16.50)
	Rihaniya	9	12.29±1.5 (9.00–14.10)	12	12.17±2.38 (7.60–15.40)

	*P* for interaction (Age × Sex × Village)		0.305		0.898

Data presented as means±SD (range). *N* indicates the number of participants. Interaction *P*-values were obtained from multivariable regression models including age and sex as continuous variables.

**Figure 1:**
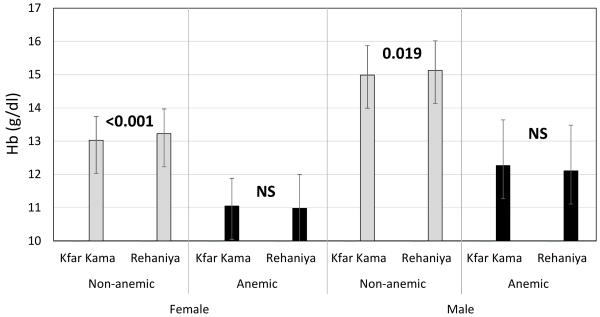
Comparison of Residence-related Hb Differences Between Non-anemic and Anemic Subjects. Results are presented as means±SD. Significance was determined as described above; NS, non-significant. For more details, see Table 3.

Given the overall difference in Hb between older versus younger populations (Table 2), we investigated whether anemia might contribute to altitude-related differences. As shown in Figure 1 and Table 3, individuals with anemia^16^ exhibited distinct altitude-related differences compared to non-anemic subjects. Specifically, a consistent, significant, albeit modest, altitude-related elevation of Hb and RBC count was observed in non-anemic cohorts. The MCV showed a minor decrease in non-anemic males related to altitude, while the MCH showed a minor decrease in non-anemic subjects of both sexes. In contrast, altitude-associated variations in blood Hb were lower in the anemic cohorts from both villages. This minimal fluctuation was accompanied by a significantly elevated RBC count, a strong decrease in MCV, and a moderate decrease in MCH among anemic residents at higher altitudes. Furthermore, the positive correlation of Hb levels with MCV and MCH, and their inverse correlation with red cell distribution width values, may suggest that iron deficiency is a primary cause of anemia in this population. Across all cohorts (non-anemic and anemic), we consistently observed a decrease in MCV in Rihaniya compared to the Kfar Kama cohorts. The HCT and MCHC fluctuated in accordance with differences in RBC count, Hb, and MCV.

## DISCUSSION

To our knowledge, this is the first study to investigate the influence of low (209 m.a.s.l. for Kfar Kama village) to moderate altitudes (674 m.a.s.l. for Rihaniya village) on RBC counts within a distinct, ethnically preserved community. Our analysis of 2,517 samples from the national CHS database provided a robust and representative dataset. The data collection strategy described herein, the substantial sample size, and the 2.5-year data collection period minimized the potential impact of data from isolated events like acute illness or pandemics.

Our primary findings revealed a clear and consistent pattern: a significant increase in residential altitude elevated Hb, RBC count, and HCT levels in male and female cohorts. These differences were accompanied by a notable decrease in MCV, together with corresponding differences in MCH and, less significantly, in MCHC. Conversely, most RBC properties in anemic participants showed minimal differences, suggesting a different physiological response to altitude adaptation in this group. The cause of this different response needs further investigation. As part of this study, we collected serum iron, transferrin, and ferritin data; however, the number of individuals who underwent these tests was extremely low and not sufficient for reliable analysis.

Comparing our results with existing literature is challenging due to the limited number of large-scale studies on the effects of low to moderate altitude on blood parameters. Despite observations over 110 years suggesting that even minor altitude differences can influence Hb levels,^17^ robust statistical conclusions from large cohorts remain scarce. Exceptions include studies by Staub et al. on 111,000 young Swiss men^9^ and Mizuta et al. on over 3 million Japanese samples.^11^ Both studies, conducted at 200–2000 and 0–800 m.a.s.l., respectively, showed an increase in Hb and associated parameters with increasing altitude, a trend consistent with what we observed in equivalent age groups. Both studies included subjects living at a wide range of altitude, while our study analyzed data from two villages with just 465 m altitude difference in a homogeneous ethnic population that represented a significant percentage of the inhabitants.

It is important to note that these referenced studies primarily focused on relatively younger and middle-aged adult populations who are generally healthier than older individuals.^18^ While our data showed an average decrease in Hb with increasing age across all altitude groups, the general trend of an altitude-associated Hb rise persisted. A recent modeling study proposed a nearly linear increase of 0.3 g/dL for every 500-meter increase in altitude between 1 and 2000 m.a.s.l.^19^ Our data demonstrated a similar average difference of ~0.2 g/dL per 500 meters, with the effect varying by sex, age, and anemia status.

**Table 3:** Interrelated Effect of the Residential Altitude and Anemia State on Hb Concentration.

Sex	Anemia Status	Place of Residence	*N*	Hb (g/dL)	RBC (10^6^/μL)	HCT (%)	MCV (fL)	MCH (pg)	MCHC (g/dL)	RDW (%)
Female	Anemic	Kfar Kama	290	11.05±0.83 (7.70–11.90)	4.10±0.50 (2.33–5.77)	34.10±2.51 (22.70–38.80)	84.04±8.68 (60.70–119.90)	27.27±3.10 (17.40–38.60)	32.41±1.05 (28.20–35.90)	14.78±1.73 (11.70–23.00)

		Rihaniya	73	10.98±1.02 (7.40–11.90)	4.41±0.59 (3.16–5.69)	34.14±2.74 (25.60–38.70)	78.62±9.79 (58.30–96.20)	25.32±3.87 (16.80–33.00)	32.10±1.41 (28.40–36.30)	15.17±2.24 (11.90–22.60)
			β	−0.07 (0.11)	0.30 (0.05)	0.08 (0.34)	−5.28 (0.75)	−1.93 (0.27)	−0.35 (0.13)	0.43 (0.15)
			*P*	0.500	**<0.001**	0.813	**<0.001**	**<0.001**	**0.010**	**0.004**

	Non-anemic	Kfar Kama	657	13.03±0.71 (12.00–17.00)	4.53±0.32 (3.54–6.40)	39.53±2.29 (32.60–52.00)	87.45±4.52 (62.00–106.40)	28.83±1.61 (20.10–34.50)	32.98±0.99 (29.50–36.80)	13.53±1.09 (11.10–22.60)

		Rihaniya	245	13.23±0.74 (12.00–17.20)	4.64±0.36 (3.85–6.31)	40.03±2.38 (32.50–53.20)	86.51±5.14 (66.20–98.90)	28.63±1.89 (19.80–33.20)	33.09±1.11 (29.30–37.00)	13.43±0.93 (11.80–17.00)
			β	0.205 (0.05)	0.11 (0.02)	0.48 (0.17)	−1.00 (0.39)	−0.22 (0.14)	0.11 (0.07)	−0.102 (0.080)
			*P*	**<0.001**	**<0.001**	**0.006**	**0.009**	0.110	0.103	0.203

Male	Anemic	Kfar Kama	122	12.27±1.37 (5.70–13.40)	4.35±0.65 (2.16–6.20)	37.64±3.99 (17.80–42.20)	87.28±7.67 (62.40–110.30)	28.46±2.84 (20.40–35.30)	32.59±1.15 (29.20–35.20)	14.45±1.73 (12.10–22.40)

		Rihaniya	44	12.11±1.37 (7.60–13.40)	4.77±0.72 (3.64–6.65)	37.65±3.57 (27.20–41.90)	79.94±9.86 (57.50–94.60)	25.73±3.71 (18.80 – 30.90)	32.11±1.30 (27.90–34.30)	15.02±1.64 (12.30–18.90)
			β	−0.17 (0.15)	0.39 (0.07)	−0.00 (0.47)	−6.83 (1.03)	−2.57 (0.37)	−0.48 (0.19)	0.59 (0.21)
			*P*	0.266	**<0.001**	0.997	**<0.001**	**<0.001**	**0.010**	**0.004**

	Non-anemic	Kfar Kama	657	14.99±0.89 (13.50–18.10)	5.08±0.37 (4.03–7.20)	44.93±2.79 (38.10–53.70)	88.63±4.59 (63.20–104.20)	29.57±1.58 (20.00–34.80)	33.37±1.07 (30.20–37.00)	13.34±0.95 (11.50–24.40)

		Rihaniya	245	15.13±0.89 (13.50–18.70)	5.18±0.44 (4.29–7.52)	45.36±2.78 (39.00–55.90)	87.91±5.69 (66.30–109.70)	29.35±1.97 (21.20–35.50)	33.39±1.16 (30.40–36.70)	13.30±0.82 (11.20–17.10)
			β	0.14 (0.06)	0.10 (0.03)	0.42 (0.19)	−0.75 (0.42)	−0.23 (0.15)	0.01 (0.07)	−0.04 (0.08)
			*P*	**0.019**	**<0.001**	**0.030**	0.078	0.136	0.814	0.638

Data presented as means±SD (range). Regression coefficients β (with standard error) were derived from a multivariable linear regression model adjusted for the interaction between sex, anemia status, and residence (village). Statistical significance was set at *P*≤0.05 and is marked in bold.

Hb, hemoglobin; HCT, hematocrit; MCHC, mean corpuscular hemoglobin concentration; MCV, mean corpuscular volume; *N*, number of participants; RBC, red blood cell count; RDW, red cell distribution width.

What factors could account for this correlation between Hb levels and low altitude? Previous research has identified a negative linear relationship between barometric pressure and arterial partial pressure of oxygen even at low altitude.^20^ The cellular oxygen-sensing mechanism, primarily the prolyl-hydroxylase-2 (PHD-2)–hypoxia-inducible factor-2 (HIF-2)–erythropoietin (EPO) axis, offers a compelling explanation.^21^ Staub et al. proposed that this sensor is remarkably precise, capable of detecting subtle differences in oxygen availability and subsequently increasing EPO production even with slight elevation.^9^ Our results strongly support this hypothesis.

In contrast to the non-anemic cohorts in our study, altitude-associated Hb variations were notably blunted in both anemic men and women. While β-thalassemia has been documented in other Circassian communities,^22^ there are no known cases of major hemoglobinopathies (e.g. sickle cell anemia or β-thalassemia) among Israeli Circassians (from AK, personal communication). Therefore, we can hypothesize that iron deficiency is the predominant cause of anemia in this population. We observed that while anemic individuals residing in the moderately higher-altitude village of Rihaniya (674 m.a.s.l.) had a small but significant elevation in RBC count compared to those in Kfar Kama (209 m.a.s.l.), their Hb levels differed minimally. This observation can be primarily attributed to abnormalities in erythropoiesis. While erythropoietin stimulation likely increased RBC production at higher altitudes, the underlying iron deficiency likely limited adequate Hb synthesis. The markedly lower MCV and MCH observed in anemic residents of higher altitudes further supports this hypothesis.

## LIMITATIONS

This study has several limitations that should be addressed in future research. First, we relied solely on the officially reported place of permanent residence and lacked data on the duration of participants’ residency. Hence, we could not fully account for individual acclimatization states, which is particularly relevant for mobile populations like students or soldiers—although based on the characteristics of this particular ethnic group, we can assume that the mobility rate was minimal. Second, we were unable to clarify the contribution of smoking, a known confounding factor for Hb adjustment.^19^ The average smoking rates in Israeli men (31%) and women (17%) are comparable to those in the Israeli Circassian community,^3^,^23^ and we anticipated that the distribution of smokers would be proportionally similar across both altitude groups in such a large cohort. Third, we did not investigate the role of specific job profiles (e.g. agricultural workers) that might involve varying levels of physical exertion or environmental exposure.

## CONCLUSIONS

This investigation of CBC data for the adult isolated Israeli Circassian population reveals that even minor differences in residential altitude (465 m) can strongly influence the Hb and RBC parameters. The observed alterations appear to be also dependent on the anemic status of the individuals. While further research is needed to fully elucidate the underlying mechanisms, our findings provide new data on the physiological impact of altitude on human health, particularly at low elevations, and suggest that daily iron requirements may vary with altitude of residence.

## Abbreviations

CBC: complete blood count
CHS: Clalit Health Services
Hb: hemoglobin
HCT: hematocrit
HMO: health maintenance organization
m.a.s.l.: meters above sea level
MCH: mean corpuscular hemoglobin
MCHC: mean corpuscular hemoglobin concentration
MCV: mean corpuscular volume
RBC: red blood cell count
